# Characterization of Notch1 Antibodies That Inhibit Signaling of Both Normal and Mutated Notch1 Receptors

**DOI:** 10.1371/journal.pone.0009094

**Published:** 2010-02-08

**Authors:** Miguel Aste-Amézaga, Ningyan Zhang, Janet E. Lineberger, Beth A. Arnold, Timothy J. Toner, Mingcheng Gu, Lingyi Huang, Salvatore Vitelli, Kim T. Vo, Peter Haytko, Jing Zhang Zhao, Frederic Baleydier, Sarah L'Heureux, Hongfang Wang, Wendy R. Gordon, Elizabeth Thoryk, Marie Blanke Andrawes, Kittichoat Tiyanont, Kimberly Stegmaier, Giovanni Roti, Kenneth N. Ross, Laura L. Franlin, Hui Wang, Fubao Wang, Michael Chastain, Andrew J. Bett, Laurent P. Audoly, Jon C. Aster, Stephen C. Blacklow, Hans E. Huber

**Affiliations:** 1 Department of Biologics Research, Merck Research Laboratories, West Point, Pennsylvania, United States of America; 2 Department of Vaccines, Merck Research Laboratories, West Point, Pennsylvania, United States of America; 3 Department of Molecular Profiling and Pharmacology, Merck Research Laboratories, West Point, Pennsylvania, United States of America; 4 Department of Pathology, Brigham and Women's Hospital, Boston, Massachusetts, United States of America; 5 Department of Pediatric Oncology, Dana Farber Cancer Institute, Boston, Massachusetts, United States of America; 6 Cancer Program, Broad Institute, Cambridge, Massachusetts, United States of America; National Cancer Institute, United States of America

## Abstract

**Background:**

Notch receptors normally play a key role in guiding a variety of cell fate decisions during development and differentiation of metazoan organisms. On the other hand, dysregulation of Notch1 signaling is associated with many different types of cancer as well as tumor angiogenesis, making Notch1 a potential therapeutic target.

**Principal Findings:**

Here we report the *in vitro* activities of inhibitory Notch1 monoclonal antibodies derived from cell-based and solid-phase screening of a phage display library. Two classes of antibodies were found, one directed against the EGF-repeat region that encompasses the ligand-binding domain (LBD), and the second directed against the activation switch of the receptor, the Notch negative regulatory region (NRR). The antibodies are selective for Notch1, inhibiting Jag2-dependent signaling by Notch1 but not by Notch 2 and 3 in reporter gene assays, with EC_50_ values as low as 5±3 nM and 0.13±0.09 nM for the LBD and NRR antibodies, respectively, and fail to recognize Notch4. While more potent, NRR antibodies are incomplete antagonists of Notch1 signaling. The antagonistic activity of LBD, but not NRR, antibodies is strongly dependent on the activating ligand. Both LBD and NRR antibodies bind to Notch1 on human tumor cell lines and inhibit the expression of sentinel Notch target genes, including *HES1*, *HES5*, and *DTX1*. NRR antibodies also strongly inhibit ligand-independent signaling in heterologous cells transiently expressing Notch1 receptors with diverse NRR “class I” point mutations, the most common type of mutation found in human T-cell acute lymphoblastic leukemia (T-ALL). In contrast, NRR antibodies failed to antagonize Notch1 receptors bearing rare “class II” or “class III” mutations, in which amino acid insertions generate a duplicated or constitutively sensitive metalloprotease cleavage site. Signaling in T-ALL cell lines bearing class I mutations is partially refractory to inhibitory antibodies as compared to cell-penetrating gamma-secretase inhibitors.

**Conclusions/Significance:**

Antibodies that compete with Notch1 ligand binding or that bind to the negative regulatory region can act as potent inhibitors of Notch1 signaling. These antibodies may have clinical utility for conditions in which inhibition of signaling by wild-type Notch1 is desired, but are likely to be of limited value for treatment of T-ALLs associated with aberrant Notch1 activation.

## Introduction

Notch signals normally participate in a variety of cellular processes, including cell fate specification, differentiation, proliferation, apoptosis, migration, and angiogenesis [1]. The four mammalian Notch receptors (Notch1-4) all have a similar modular domain organization. The extracellular domain contains a series of epidermal growth factor (EGF)-like repeats that participate in binding to ligands [2], followed by a negative regulatory domain (NRR) that, in the absence of ligand, maintains the receptor in a protease-resistant conformation [3], [4]. During trafficking to the cell surface, the NRR is clipped by a furin-like protease at a site called S1 [5], dividing Notch into two subunits that are held together by contacts in the N-terminal and C-terminal portions of the NRR. The intracellular portion of Notch (ICN) contains RAM [6] and ankyrin-repeat domains that both participate in binding to the DNA-binding factor CSL [for CBF-1/Su(H)/Lag1] [7], as well as nuclear localization sequences and a C-terminal PEST degron [8].

Activation of Notch receptors is normally induced by binding of Jagged [9], [10] or Delta-like [11]–[13] ligands expressed on neighboring cells, which initiates a series of additional proteolytic cleavages. The first is catalyzed by a metalloprotease of the ADAM (a disintegrin and metalloprotease) family [14], [15] and occurs at a site called S2, which lies within the NRR just external to the transmembrane domain. This primes Notch for additional cleavages within the transmembrane domain that are carried out by the multiprotein membrane complex γ-secretase [16]. The final cleavage liberates ICN from the membrane, allowing it to enter the nucleus and activate the transcription of Notch-responsive genes (e.g., *HES1*, *HES5*, *NRARP*, *Deltex1* (*DTX1*), c-*MYC*). This depends on binding of ICN to the transcription factor CSL [7], [17], [18] and recruitment of Mastermind-like coactivators [19]–[22]. Post-translational modification events, such as glycosylation of the extracellular domains of both receptor and ligands also play an important role in Notch-ligand interactions [23], [24], and such modifications may play a part in tissue-specific responses to various ligands [25].

In addition to its developmental roles, dysregulation of Notch signaling is associated with a number of different cancers. The clearest example is T-cell acute lymphoblastic leukemia/lymphoma (T-ALL, see below), in which activating mutations in the NRR and/or the PEST domain of Notch1 are found in over 50% of cases. Increases in Notch signaling, perhaps induced by ligand-mediated activation, have also been associated with breast, colon, ovarian, and lung cancer [26]–[30]. For example, co-expression of Notch1 and Jag1 has been associated with poor outcomes in patients with breast cancer [31]. Delta-like-4 signaling through Notch1 regulates the formation of tip cells during angiogenesis [32] and is also likely to play an important role in pathological angiogenesis [33], making it a promising therapeutic target [34].

The discovery of gain-of-function Notch1 mutations in 55–60% of human primary T-ALL samples [26], including all of the major T-ALL subtypes, greatly expanded the known role of Notch1 in this disease, moving it to the center of T-ALL pathogenesis. The most common leukemogenic Notch1 mutations (35–40% of tumors) lie in the “heterodimerization domain” (HD) of the NRR and lead to ligand-independent Notch signaling activity [35], [36]. Mutations that result in deletion of the PEST degron (20–30% of tumors) are also frequent in T-ALL and cause a synergistic increase in Notch signaling when aligned in *cis* with HD mutations in the same Notch1 allele [35]–[37]. Notch1 signaling drives the growth of T-ALL cells [38], [39], making it an attractive target for rational pharmacological intervention.

A number of different strategies [34] are in development to inhibit Notch signaling for therapeutic purposes. One approach is to block the proteolytic release of intracellular Notch from the membrane by treatment with inhibitors of gamma secretase (GSIs). In a number of tumor cell lines carrying HD domain mutations, blocking proteolytic activation with GSIs triggers cell-cycle arrest and variable degrees of apoptosis [40], [41]. However, the poor selectivity of GSIs, which inhibit the proteolysis of all four Notch receptors, and the processing of an expanding list of other substrates by gamma secretase [16], [42], [43], constitute significant potential limitations for this class of anti-tumor agents. Studies in animal models using the GSI LY 411,575 have shown significant dose-limiting toxicity in the intestine [44]. The toxic effects of GSIs in mice appear to result from simultaneous inhibition of Notch1 and Notch2 [29], [45], which leads to the accumulation of secretory cells at the expense of absorptive enterocytes. Clinical trials with the GSI LY450139 in Alzheimer's disease patients also identified diarrhea as the most frequent adverse effect in human phase I studies [46].

An alternative route that may overcome the toxicity associated with GSIs is selective targeting of Notch1 with inhibitory antibodies. In support of this approach, antibodies capable of selectively modulating Notch3 signaling have been reported recently [47]. The most potent inhibitory antibodies are directed against the NRR and are proposed to stabilize the autoinhibited form of the receptor [47].

In this study, we report the *in vitro* activities of inhibitory Notch1 monoclonal antibodies derived from cell-based and solid-phase screening of a phage display library. Two different classes of antibodies were identified. One class is ligand-competitive, being directed against the EGF-repeat region of the receptor that encompasses the ligand-binding domain (LBD), and the second is allosteric, being directed against the NRR region. Both classes of antibodies are selective for Notch1, bind Notch1 on the surface of human tumor cell lines, and inhibit ligand-induced expression of Notch target genes in cell lines expressing wild-type Notch1 receptors. NRR-targeting antibodies are also capable of recognizing and inhibiting Notch1 receptors bearing “class 1” NRR mutations, but are less effective in inhibiting Notch1 activation in T-ALL cells than GSIs. These findings have implications for selective targeting of normal and mutated Notch1 receptors with antibodies as well as our understanding of Notch1 receptor activation in T-ALL cells.

## Materials and Methods

### Cell Culture and Reagents

Cancer cell lines (LS-1034, BxPC3, Colo_205, and TALL-1) purchased from ATCC (Manassas, VA) were maintained at 37°C under 5% CO_2_ in RPMI 1640 (Invitrogen, Carlsbad, CA) supplemented with 10% heat-inactivated (HI) FBS (Hyclone, Logan, Utah), 2 mM L-glutamine (Invitrogen) and 1× Pen-Strep (Mediatech, Herndon, VA). T-REX™-293 and Flp-In™ -3T3 cell lines purchased from Invitrogen were maintained at 37°C under 5% CO_2_ in Dulbecco modified Eagle medium (DMEM) with high glucose (Invitrogen) supplemented with 10% HI FBS (Hyclone), 2 mM L-glutamine (Invitrogen), and 1× Pen-Strep (Mediatech). For the ligand stimulation assays, cells were resuspended in DMEM high Glucose medium without phenol red and supplemented only with 10% HI FBS (Hyclone).

### Construction of cDNAs and Generation of Stable Cell Lines

Cell lines stably expressing either full-length wild-type or chimeric Notch receptors or Notch ligands were generated to test the binding and potency of Notch antibodies. The human and mouse (only Notch1) full-length cDNA sequences coding for Notch1, 2, and 3, Jag1, and DLL1 were chemically synthesized by DNA2.0 Technologies (Menlo Park, CA). The cDNA encoding DLL4 was amplified by RT-PCR from Colo_205 cells following described protocols [48]. Chimeric human Notch receptors (Notch1, 2, and 3) were created by inserting the sequence encoding the DNA binding domain of Gal4 into a Notch cDNA previously deleted of the sequence encoding most of the RAM domain and the ankyrin repeat domain as described [35]. Each of these cDNAs were cloned into the pcDNA5/FRT/TO vector (Invitrogen) and co-transfected with pOG44 (Invitrogen), a plasmid encoding Flp recombinase, into T-REX-293 cells (human wild-type Notch receptors), T-REX-U2OS cells (mouse wild-type Notch1, chimeric receptors), or 3T3 Flp-In cells (Notch ligands) using Fugene6 (Roche, Indianapolis, IN) following the manufacturer's protocols. Cell lines with stably integrated cDNAs were selected with hygromycin (100 µg/ml) (Mediatech). Expression levels of the Notch proteins were assessed by Western blot and flow cytometry. The T-REX-U2OS and -293 cells express the Tet repressor (for tight regulation of gene expression), and also contain a single genomic FRT (Flp recombinase target) site, which permits creation of isogenic recombinants containing a single transgene under the control of tetracycline.

### Luciferase Reporter Assays

Assays were performed as described [35]. Stable Flp-In U2OS cells bearing isogenic transgenes encoding Notch1-, 2-, or 3-GAL4 chimeric receptors were transiently transfected with a pFR-Luc reporter plasmid (Stratagene, Cedar Crest, TX) containing five copies of the Gal4 binding site (5X-upstream activation sequence (UAS)). After 24 hr, cells were treated with doxycycline (2 µg/ml) (Sigma-Aldrich, St. Louis, MO) in the presence or absence of Notch antibodies and IgG control, and overlaid at a 1∶1 ratio onto Flp-In-3T3 cells stably expressing Notch ligand or parental Flp-In-3T3 cells seeded in 96-well plates. Luciferase reporter activities were measured after an additional 24 hr in whole cell lysates using the Bright-Glo assay kit (Promega, Madison, WI) and a VictorLight luminometer (Perkin-Elmer, Waltham, MA). The potency of the antibodies was determined with serial dilutions that generate sigmoid curves. IC_50_ values were determined using a 3-parameter logistic curve fit with maximum values arbitrarily fixed at 100%. In the mouse Notch1 reporter assay, Notch activity in cells stably expressing the mouse full-length wild-type Notch1 receptor was determined as described above using a luciferase reporter gene containing four copies of the CSL binding site (4XCSL-luciferase reporter gene). To test ligand-independent activation of Notch1 signaling generated by receptors bearing specific T-ALL mutations, T-REX-U2OS cells were transiently transfected with full-length Notch1 DNA constructs bearing those mutations and the 4XCSL-luciferase reporter gene. Cells were then treated with antibodies (10 µg/ml) and luciferase activity measured after 24 h as described above. In these experiments, T-REX-U2OS cells transiently transfected with the full-length wild-type Notch1 receptor were used as the baseline control.

### Ligand Competition Assay

Ligand-competitive binding of antibodies was measured by Notch1 extracellular domain (ECD) displacement in a dissociation-enhanced time resolved fluorometric assay (DELFIA). Briefly, Maxisorp 96-well plates were coated with Notch ligand DLL4 (100 µl/well, R&D Systems, Minneapolis, MN) at 2 µg/ml in D-PBS (GIBCO-14080) and incubated at 4°C overnight. The coated plates were blocked with 5% BSA in D-PBS. Notch1 ECD-Fc fusion protein (Notch1 amino acids Ala19-Gln526) (R&D Systems) was pre-labeled with europium (Eu) reagent according to the manufacturer's procedure (PerkinElmer). Serially diluted Notch1 monoclonal antibodies were preincubated with a fixed amount (0.5 µg/ml) of Eu-labeled Notch1 ECD-Fc for 2 hr with shaking, followed by addition to the DLL4 ligand-coated plates. After incubation at room temperature for 1 hour with slow shaking, the plates were washed and DELFIA® Enhancement solution (Perkin Elmer) (100 µl/well) added. Fluorescence signals were read after a 5-min incubation in a Victor3-V plate reader (Perkin Elmer) at excitation/emission wavelengths of 340/615nm.

### Flow Cytometry

Flow cytometric detection of Notch1 was performed with doxycycline-inducible T-REX-293 cells stably expressing the full-length Notch1, -2, or -3 receptors, respectively; or the human cancer cell lines LS-1034, BxPC3, Colo_205, and TALL-1. T-REX-293 cells were treated with 2 µg/ml doxycycline (Sigma-Aldrich) for two days to induce Notch expression before collecting them for staining. Flow cytometric detection of Notch ligands was performed in Flp-In-3T3 cells stably expressing Jag1, Jag2, and DLL1 ligands, respectively, using primary antibodies purchased from R&D Systems. Cells were harvested from tissue culture flasks using trypsin (Mediatech) and resuspended in PBS with 2% fetal bovine serum (FBS; Hyclone) (FBS/PBS). Cells (1×10^6^) were incubated with 1 µg of each antibody for 40 minutes at 4°C, followed by washing and resuspension in 0.1 ml of FBS/PBS containing 1 µg of phycoerythrin-conjugated secondary antibodies for 30 minutes at 4°C. Cells were then washed, resuspended in 0.34 ml PBS with 1% formaldehyde (Polysciences, Inc., Warrington, PA) and analyzed on a FACSCalibur (Becton Dickinson, San Jose, CA). The secondary antibodies used were goat anti-human Fcγ specific F(ab')_2_ fragments (Jackson ImmunoResearch, West Grove, PA) to detect the primary anti-Notch1 Abs, donkey anti-goat IgG (H+L) specific F(ab')_2_ fragments (Jackson ImmunoResearch) to detect anti-Jag1 and anti-Jag2, and rat anti-mouse IgG κ light chain antibody to detect anti-DLL1 (BD Pharmingen, San Diego, CA).

### RNA Extraction and QRT-PCR

Parental Flp-In 3T3 cells or Jag2-expressing 3T3 cells were co-cultured at a ratio 1∶1 with LS-1034 or TALL-1 cells in the presence of 20 µg/ml of each antibody or 5 µM GSI for 19 hr. Cells were harvested and RNA was isolated using the RNeasy Mini Kit (Qiagen, Valencia, CA). cDNA was synthesized from 0.5 µg of purified RNA using the High Capacity cDNA Archive Kit (Applied Biosystems, Foster City, CA) according to the manufacturer's instructions. qPCR was performed in triplicate using 2 µl cDNA sample or control, Brilliant II QPCR Master Mix with ROX (2X) (Stratagene, Cedar Creek, TX), and the inventoried probes and primers (Applied Biosystems Assays on Demand) for human *HES1*, *HES5*, *DTX1* (Deltex1), and *GAPDH*. PCR cycling was performed at 95°C for 10 minutes to allow enzyme activation, followed by 40 cycles of 95°C for 15 seconds and 60°C for 1 minute using the Mx3005P QPCR System (Stratagene). Analysis was performed using MxPro QPCR Software version 3.0 (Stratagene).

### Proliferation Assays

Human T-ALL cell lines (TALL-1, DND-41, KOPT-K1) were cultured at 37°C under 5% CO_2_ in RPMI 1640 (Invitrogen) supplemented with 10% FBS (Hyclone), 2 mM L-glutamine (Invitrogen), 100 UI/ml penicillin G, and 100 µg/ml streptomycin (Invitrogen). For growth assays, 30 µl of cell suspension (9×10^4^ cells/ml) was seeded in 384-well white plates (Corning, Lowell, MA). Drugs and/or antibodies were added after 4 hr of pre-culture. Drugs were dissolved in DMSO and antibodies in sterile PBS. Assessment of the cell growth after 72, 96, and 120 hr of treatment was carried out by the Cell-Titer Glo luminescence assay (Promega) in an Envision Multi-label plate reader (Perkin-Elmer, Waltham, Massachusetts). The assay was done in triplicate and each experiment was repeated twice. The significance of differences in cell viability was assessed by the Student t-test. Synergism was determined by isobologram analysis of dose-response curves for cells exposed to varying concentrations of GSI (compound E, Axxora, San Diego, CA), dexamethasone (Sigma), and/or Notch1 inhibitory antibodies. When used in combination, the ratio of the two drugs was 1∶1, while the ratio of antibody to drug was 40∶1. The degree of synergism was determined by the combination index (CI) method [49], which was computed from the dose-response curves with Calcusyn version 2 software (Biosoft, Cambridge, UK). Significant synergism was defined as a CI<0.7.

### Effects of Notch1 Antibodies on Gene Expression in T-ALL Cells

In order to determine the effects of Notch1 antibodies on the Notch1-dependent gene expression signature in T-ALL cells, we first selected a set of genes that defined the Notch1 *on* versus *off* state from Affymetrix microarray expression profiling of 7 Notch1-mutated T-ALL cell lines treated in duplicate with vehicle versus the GSI compound E (500 nM) for 24 hr [39]. From a set of ∼500 genes with differences of p<0.01 by 2-sided Student's t-test, 16 sentinel genes were selected to define the Notch1 *off* signature based on mean fold changes >1.5 between the Notch1 *on* versus *off* states. Four control genes with stable expression across the two states were selected to control for well-to-well variability in total RNA: *GAPDH*, *NFX1*, *NISCH*, and *GTF*. We next adapted this signature to an assay that uses ligation-mediated amplification (LMA) and a Luminex FlexMAP fluorescent bead-based detection system. Full details of this methodology have been described elsewhere [50]. Briefly, the 20 genes were subjected to 34 cycles of amplification by LMA, yielding biotinylated PCR products containing molecular barcode sequences. These PCR products were hybridized in solution to beads dyed with unique fluorescent colors containing complementary barcode sequence. Following hybridization and staining with streptavidin-phycoerythrin (SA-PE), the beads were analyzed by dual-color flow cytometry, in which the bead color identifies the gene of interest and PE intensity the quantity of transcript. DND-41 cells were treated with control antibody (10 µg/ml) (6 replicates), NRR WC75 Notch1 antibody (10 µg/ml) (7 replicates), DMSO (0.08%) (6 replicates), or GSI (1 µM) (14 replicates) for 72 hr and then analyzed for gene expression. To normalize measurements within each experiment, expression of Notch marker genes was expressed relative to the average expression of the four control genes. We also evaluated the overall performance of the signature by calculating two summary scores combining information about all of the signature genes: the summed score and the weighted summed score. The summed score metric combined expression ratios by summing them with a sign determined by the expected direction of regulation as determined from the positive controls (GSI-treated). The weighted summed score metric is a variant of the summed score metric that combines expression ratios by summing them with a weight and sign determined by the signal-to-noise ratio of the positive control (GSI-treated) and negative controls (DMSO-treated). Signal-to-noise ratio is defined by:
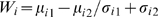
where *μ_i1_* represents the mean expression of samples from class 1 for feature *i* and *σ_i1_* represents the standard deviation of class 1 for feature *i*.

### Antibody-Mediated Immunoprecipitation of the Notch1 Ectodomain

Notch ectodomains were cloned into the pLEXm mammalian expression vector with C-terminal His_6_ tags, expressed in HEK-293T cells using a PEI-based transfection protocol and harvested after 3 days. Whole cell extracts were prepared by lysis with RIPA buffer containing 1∶250 protease inhibitors followed by centrifugation to remove cellular debris. Lysates were mixed with 5–10 µg primary antibody overnight at 4°C, then with 50 µL Protein A-agarose suspension for an additional 2 hours. Beads were then collected by centrifugation, washed 3 times with PBS, and resuspended in 50 µL 2× SDS sample buffer containing 100mM DTT. Samples were boiled for 5 minutes and run on a 5–20% Tris-Glycine gel, then transferred to a nitrocellulose membrane at 15V for 1 hour. Blots were washed twice with Tris-buffered saline, pH 7.5 (TBS) and blocked in TBS +3% BSA for 1 hour. Incubation with Penta-His antibody (1∶1000) was performed overnight in TBS +3% BSA, followed by incubation with goat α–mouse antibody (1∶10,000) in TBS +10% milk for 1 hour. Three wash steps were done following the incubation with primary antibody (two with TBS-Tween-0.2% Triton-X (TBST) and one with TBS), and four washes with TBST were done following the incubation with secondary antibody. Blots were exposed for 10–20 minutes and analyzed using an Alpha Innotech gel documentation system.

### Calcium-Dependence of Epitope Binding by Anti-NRR Antibodies

A plasmid encoding the human Notch1 NRR (residues E1446-Q1733; Genbank ID 148833507) was modified to contain a N-terminal hexahistidine tag followed by a TEV cleavage site. The Notch1 NRR precursor was prepared essentially as described for the loopout form of the Notch1 NRR [37]. The purified protein was labeled with EZ-Link NHS-PEG4-Biotin (Pierce-Thermo, Rockford, IL) according to the manufacturer's instructions. Biotinylated Notch1 NRR was then captured onto neutravidin-coated 96-well plates. Binding of the anti-NRR antibodies was allowed to proceed for one hour in Tris buffer (25 mM, pH 7.4), containing NaCl (150 mM), CaCl_2_ (5 mM), 0.05% Tween, and 0.5% BSA. Antibody binding was detected with a goat anti-human antibody conjugated to horseradish peroxidase using the fluorogenic substrate quantaBlu (Pierce-Thermo).

## Results

### Notch1 Antibodies Bind to Distinct Domains (LBD or NRR) of the Notch1 Receptor

**Figure 1:**
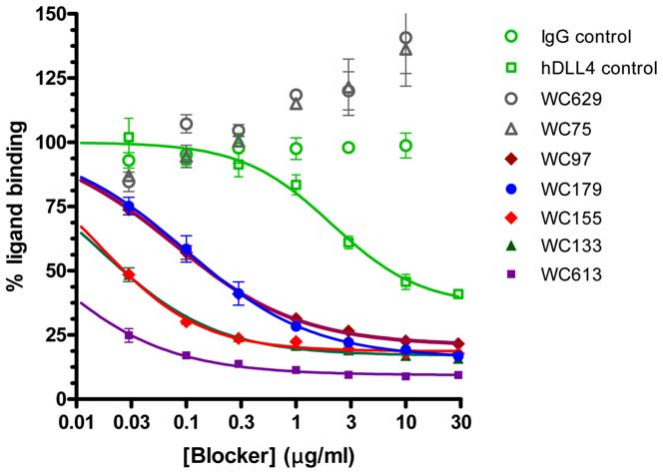
Ligand-competition by Notch1 antibodies. A panel of Notch1 antibodies was tested in a ligand competition assay (DELFIA) for binding to EGF repeats 1–13 of Notch1. This assay measures inhibition of binding of Eu-labeled Notch1 ECD-Fc fusion protein to immobilized DLL4. The ECD-Fc fusion comprises EGF repeats 1–13 (Ala19-Gln526) which includes the ligand binding domain (EGF repeats 11–13) but lacks the NRR. Human IgG and competing soluble DLL4 were used as negative and positive controls, respectively. The data were normalized with respect to the “no blocker” controls and curve fitted using a fixed 100% plateau, shared slopes and variable base lines. Error bars represent the standard deviation from triplicate values.

A total of 16 high-affinity antibodies against Notch1 were selected from a phage library, using both cell-based and recombinant protein panning approaches. The details of the panning strategy and the identification, cloning and ranking of hits by affinity will be described elsewhere. All antibodies were shown by flow cytometry to bind to full-length Notch1 overexpressed in HEK cells (Table 1 and data not shown). Since antibodies against the Notch1 extracellular domain were likely to interfere with receptor-ligand interactions, we first evaluated all antibodies in a ligand competition assay with recombinant DLL4 and the Notch1 ectodomain (EGF repeats 1–13), which includes the ligand binding domain (LBD; EGF repeats 11–13). As shown for a subset of antibodies in Figure 1, most, but not all, antibodies inhibited binding of the Notch1 ectodomain to immobilized DLL4. Fixed dilutions of antibodies were used for this initial characterization, as the intent was to classify the antibodies by mechanism rather than to establish their relative potencies. Antibodies that failed to compete with DLL4 binding, such as WC75 and WC629 (Figure 1), did not recognize EGF repeats 1–13. Instead, these antibodies were found to bind to the negative regulatory region (NRR) of Notch1 (supplemental Figure S1). In contrast, none of the ligand-competitive antibodies bound to recombinant NRR (data not shown). A total of seven antibodies (listed in Table 1) were selected for further characterization, five ligand-competitive antibodies (referred to as LBD antibodies) and two antibodies recognizing the NRR (referred to as NRR antibodies).

**Table 1:** Inhibition of Jag2-mediated Notch1 signaling by Notch1 antibodies.

Antibody	Target domain^a^	Notch1 Binding^b^	Notch1 inhibition	
			EC_50_ (nM)^c^	% maximal
WC613	LBD	++	5 (±3)	96 (±1)
WC133		++	10 (±5)	95 (±3)
WC155		++	57 (±37)	>80
WC179		+	43 (±16)	>70
WC97		+	170 (±25)	>70
WC75	NRR	++++	0.13 (±0.09)	75 (±9)
WC629		+++	6 (±2)	70 (±3)

a LBD: EGF-like repeats (1–13) encompassing the ligand-binding domain; NRR: Negative regulatory region.

b FACS score on Notch1 expressing HEK cells: MFI<100 (+); MFI 100–500 (++); MFI 501–1000 (+++); MFI>1000 (++++); MFI = Mean Fluorescence Intensity.

c Co-culture assay with Notch1-Gal4 T-REX-U2OS cells transfected with UAS-luciferase reporter and Flp-In-3T3-Jag2 cells; normalized to signal obtained with Flp-In-3T3 parental cell line; average and standard deviation from at least 4 independent experiments.

### LBD and NRR Antibodies Are Specific Notch1 Antagonists

To evaluate the functional activity of the LBD and NRR antibodies, we generated a panel of stable T-REX-U2OS cell lines expressing Notch1-, Notch2-, or Notch3-Gal4 fusion receptors. Notch signaling in these reporter cell lines was monitored by transient transfection of a Gal4-luciferase reporter plasmid. To activate Notch signaling, reporter cell lines were co-cultured with 3T3 cell lines stably overexpressing various Notch ligands.

**Figure 2:**
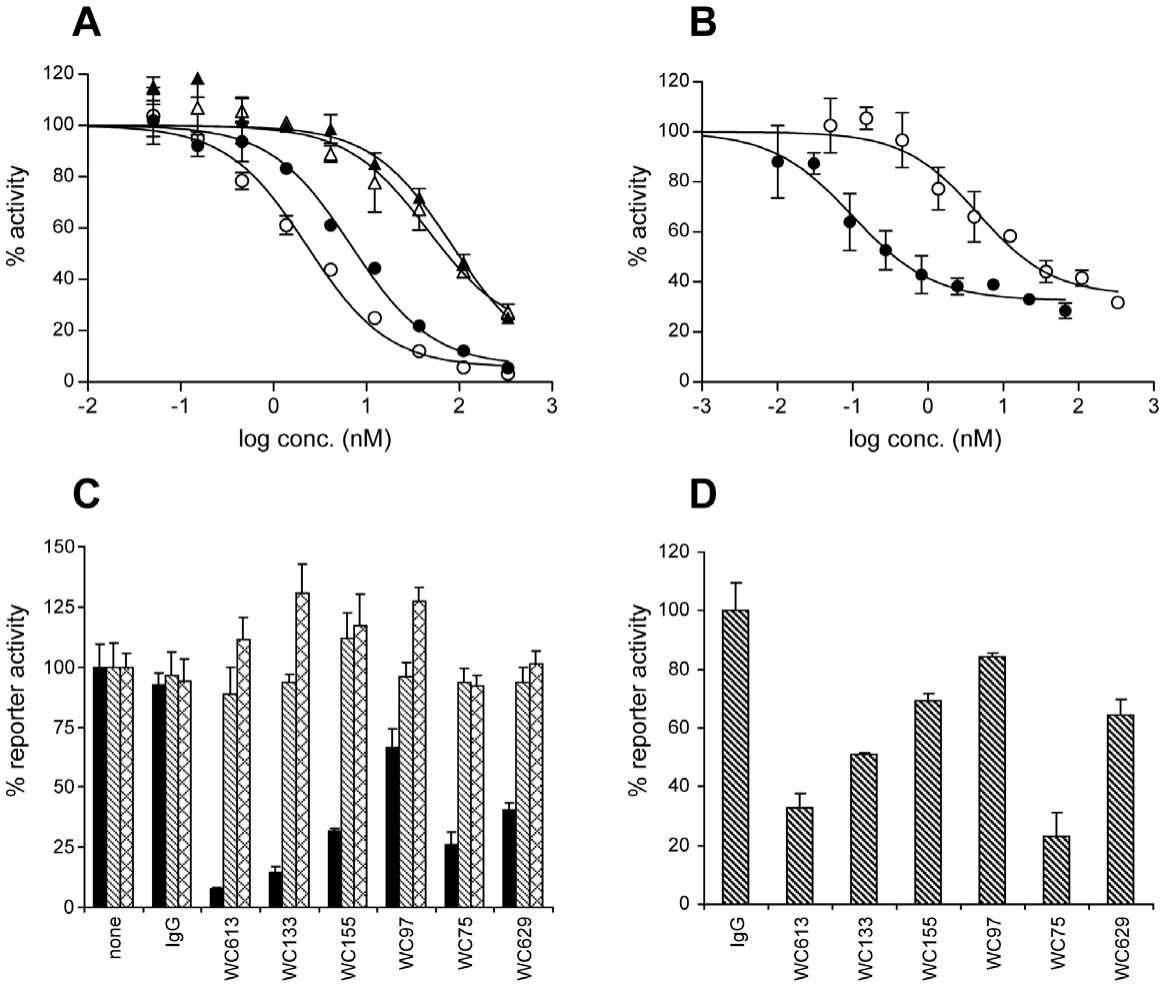
Inhibition of Jag2-dependent Notch signaling by Notch1 antibodies. Notch1 reporter activity was measured in co-culture assays with T-REX-U2OS Notch1-Gal4 reporter cells and Flp-In-3T3 cells expressing human Jag2. Representative examples are shown for each of the LBD and NRR antibodies. Error bars represent the standard deviation from triplicate values. (A) Inhibition of Notch1-Gal4 signaling by LBD antibodies WC613 (open circles), WC133 (closed circles), WC179 (open triangles), and WC97 (closed triangles). (B) Inhibition of Notch1-Gal4 signaling by NRR antibodies WC75 (closed circles) and WC629 (open circles). (C) The Notch isoform specificity of Notch1 antibodies was tested in co-culture assays with T-REX-U2OS Notch-Gal4 reporter cells (human Notch1, 2, and 3) and Flp-In-3T3 cells expressing human Jag2. NRR and LBD antibodies were used at a fixed antibody concentration of 167 nM. Reporter cell lines used: hNotch1-Gal4 (black bars), hNotch2-Gal4 (lined bars), hNotch3-Gal4 (cross-hatched bars). The activity of a UAS-luciferase reporter transiently expressed in the T-REX-U2OS Notch-Gal4 cells was normalized to untreated controls. IgG isotype controls are shown. Error bars represent standard deviation. (D) The species specificity of Notch1 antibodies was tested in co-culture assays with T-REX-U2OS cells expressing wild-type mouse Notch1 and Flp-In-3T3 cells expressing human Jag2. NRR and LBD antibodies were used at a fixed concentration of 167 nM. The activity of a 4xCSL-luciferase reporter transiently expressed in the T-REX-U2OS cells was normalized against the non-specific IgG control. Error bars represent standard deviation.

The five LBD and two NRR antibodies were characterized for their effect on Jag2-induced Notch signaling, using a well-characterized NIH 3T3 cell line expressing Jag-2 [51]–[52]. The ligand-competitive LBD antibodies were able to block Jag2-stimulated Notch1 activity completely (Figure 2A and Table 1). The basal reporter activity in the presence of parental Flp-In-3T3 cells (no ligand expression) was neither significantly inhibited nor stimulated by these antibodies (data not shown), indicating that, despite their potential to crosslink Notch1, free LBD antibodies do not have agonistic activity. The NRR-specific antibodies were also potent antagonists of Jag2-dependent Notch1 signaling (Figure 2B and Table 1), presumably through allosteric stabilization of the NRR domain in a metalloprotease-resistant autoinhibited conformation. The generally greater potency of the NRR antibodies as compared to the LBD antibodies correlated with a higher affinity for Notch1-expressing cells, as assessed by flow cytometry (Table 1). However, at saturating concentrations, the NRR antibodies maximally inhibited Notch1 signaling by 70 to 80%, whereas LBD antibodies were able to completely inhibit the Notch1 activation by Jag2. As seen with the LBD antibodies, the NRR-binders did not exhibit detectable agonist activity in co-culture assays using parental 3T3 Flp-in cells.

The specificity of both classes of antibodies for Notch1 as opposed to Notch2 or Notch3 was evaluated in reporter assays with Notch-Gal4 fusion receptors. Neither LBD nor NRR antibodies significantly inhibited ligand-stimulated Notch2-Gal4 and Notch3-Gal4 signaling (Figure 2C). Species cross-reactivity was tested in T-REX-U2OS cells stably expressing murine Notch1 and transiently transfected with a CSL-luciferase reporter. In co-culture experiments with 3T3 cells expressing human Jag2, greater than 50% inhibition of mouse Notch1 was seen with several of the antibodies at 167 nM (Figure 2D), suggesting that future efficacy and tolerability studies of these antibodies can be conducted in mouse models.

Because we have been unable to create Notch4 reporter lines that generate a luciferase signal in response to ligand stimulation, we tested the antibodies for specificity toward Notch1 as opposed to Notch4 by comparing the ability of representative LBD and NRR antibodies to immunoprecipitate the Notch1 and Notch4 extracellular domains. Western blot analysis (supplemental Figure S2) showed that the allosteric antibody WC75 and the ligand competitive antibody WC613 both immunoprecipitated Notch1 but not Notch4.

### Ligand Dependence of Inhibitory LBD and NRR Antibodies

**Figure 3:**
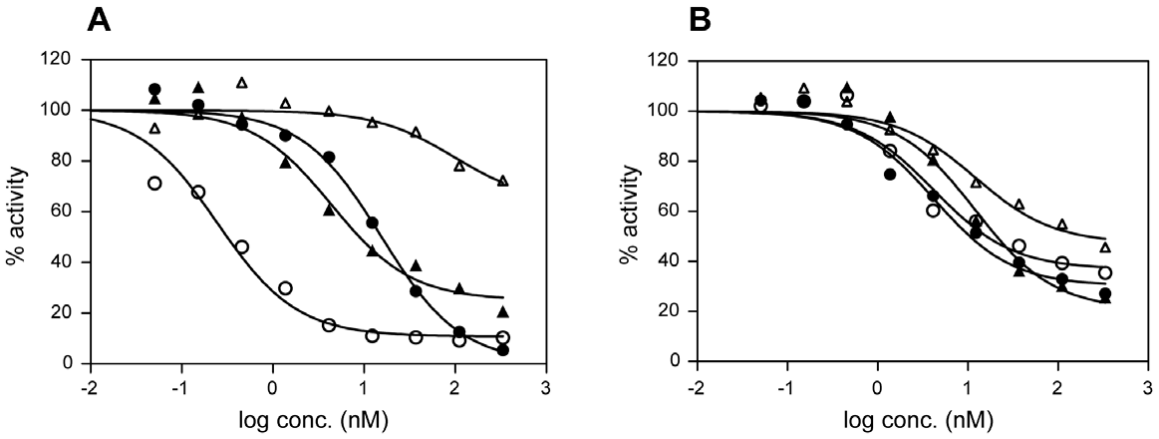
Ligand-dependence of Notch1 inhibition by LBD and NRR antibodies. Notch1 signaling in T-REX-U2OS Notch1-Gal4 cells was stimulated by co-culture with Flp-In-3T3 cells expressing Jag2 (closed circles), Jag1 (open circles), DLL1 (closed triangles), or DLL4 (open triangles). The activity of a UAS-luciferase reporter transiently expressed in the T-REX-U2OS Notch-Gal4 cells was normalized to untreated controls. Representative examples of Notch1-Gal4 reporter inhibition are shown for LBD antibody WC613 (A) and for NRR antibody WC629 (B).

Based on current models of Notch receptor activation, the inhibitory activities of LBD antibodies were expected to show a stronger dependence on the activating Notch1 ligand than NRR antibodies, which are not ligand-competitive (Figure 1). We therefore compared the ability of these two classes of antibodies to inhibit Notch1 signaling in co-culture assays with 3T3 cells stably expressing Jag1, Jag2, DLL1 or DLL4. Expression of Jag1, Jag2, and DLL1 was confirmed by flow cytometry using specific antibodies in each of the stable 3T3 cell lines (data not shown). It was not possible to determine the levels of DLL4 by flow cytometry due to lack of an appropriate antibody, but luciferase reporter assays using 3T3-DLL4 cells confirmed the ability of DLL4 to strongly activate Notch signaling. As expected, the antagonistic activity of LBD antibody WC613 was strongly dependent on the particular ligand-expressing cell line used to activate Notch1 (Figure 3A). 3T3-Jag1 mediated signaling was most sensitive to inhibition, while 3T3-DLL4 signaling was most resistant. The same rank order was observed with the other LBD antibodies (Table 2). While it is tempting to speculate, the variation in EC_50_ values cannot be solely attributed to the identity of the particular ligand since we were not able to quantify the ligand levels on the various 3T3 cell lines in absolute terms. In contrast to the LBD antibodies, the activities of both NRR antibodies were minimally dependent on the nature of the ligand-expressing cell line and both antibodies inhibited DLL4 signaling, albeit to a maximum of 50% to 60% (Figure 3B, Table 2). These data are consistent with the current models of ligand-dependent Notch receptor activation, i.e., that LBD antibodies compete with ligands for access to the Notch1 LBD, whereas NRR antibodies allosterically inhibit ligand-induced conformation changes in the NRR.

**Table 2:** Inhibition of signaling of various Notch1-ligand pairs by Notch1 antibodies.

Antibody	Domain^a^	EC_50_ (nM)^b^			
		Jag2	Jag1	DLL1	DLL4
WC613	LBD	5 (±3)	0.3 (±0.2)	5 (±2)	>330
WC133		10 (±5)	0.7 (±0.1)	7 (±3)	>330
WC155		57 (±37)	1.3 (±1)	48 (±6)	>330
WC179		43 (±16)	3.4 (±3)	28 (±16)	>330
WC97		170 (±25)	4 (±2)	18 (±10)	>330
WC75	NRR	0.13 (±0.09)	0.1 (±0.1)	0.3 (±0.3)	0.32 (±0.3)
WC629		6 (±2)	2 (±2)	6 (±4)	∼50^c^

a LBD: EGF-like repeats (1–13) encompassing the ligand-binding domain; NRR: Negative regulatory region.

b Co-culture assay with Notch1-Gal4 T-REX-U2OS cells transfected with UAS-luciferase reporter and Flp-In-3T3 cells overexpressing the ligands Jag2, Jag1, DLL1, and DLL4, respectively; normalized to signal obtained with Flp-In-3T3 parental cell line; average and standard deviation from at least 4 independent experiments.

c Inflection points poorly defined.

### Notch1 Antibodies Modulate Notch Target Gene Expression in Cancer Cell Lines

**Figure 4:**
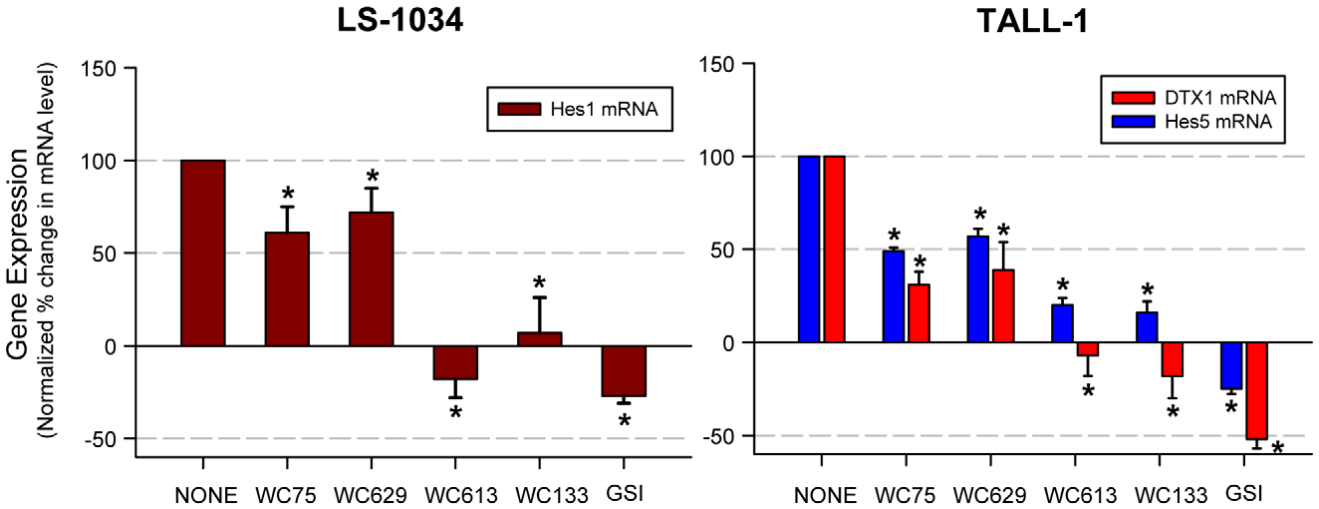
Notch1 antibodies inhibit Notch target gene expression in cancer cell lines. The ability of antibodies (20 µg/ml) or GSI (5 µM) to inhibit Notch1 target gene expression (*HES1*, *HES5*, *DTX1*) was analyzed by quantitative real time PCR (qRT-PCR) of mRNA extracted from LS-1034 or TALL-1 cancer cell lines co-cultured with Flp-In 3T3-Jag2 cells for 22 h at 37°C. qRT-PCR was performed in triplicate with the Stratagene Mx3005P (Agilent Technologies, BioCrest Manufacturing, Cedar Creek, TX). Values were normalized on the basis of GAPDH mRNA expression. Gene expression (% mRNA remaining) normalized to Jag2-dependent signal (100%) from at least four experiments is represented (error bars indicate error standard, *p<0.05).

Notch1 signaling is increased in a variety of cancers and activates downstream target genes, including *HES1*, *HES5*, *DTX1*, *NRARP*, and *c-MYC* [7], [18], [38], [53]. Two human lines expressing wild-type Notch1 receptors, the colorectal carcinoma cell line LS-1034 and the T-ALL cell line TALL-1, were used to test the ability of the LBD and NRR antibodies to modulate Notch activity. Notch signaling was induced by co-culture of these cell lines with 3T3 cells expressing Jag2. Although other Notch receptors are expressed in these cells (i.e., Notch2 and 3 was detected in LS-1034 cell lysates by Western blot, and Notch3 on the surface of TALL-1 cells by flow cytometry; data not shown), the specificity of the antibodies (unlike GSI) allows one to assess the effects of Notch1 inhibition *per se*. In LS-1034 cells, ligand-dependent transactivation of Hes1 transcription was inhibited significantly by each of the antibodies tested at saturating antibody concentrations. The LBD antibodies (e.g., WC613, WC133) almost completely inhibited Hes1 transactivation, while the NRR antibodies (WC75, WC629) were partially inhibitory (Figure 4). A similar correlation was observed in TALL-1 cells, in which the ligand-dependent transactivation of both *HES5* and *DTX1* was also inhibited by all of the antibodies tested at saturating antibody concentrations, with the LBD antibodies being more effective than the NRR antibodies (Figure 4). These results correlate with the ability of the LBD and NRR antibodies to totally or partially, respectively, inhibit ligand-dependent Notch1 activation in the reporter assays. Because other Notch family members besides Notch1 may contribute to basal and induced Notch signaling in these cells, we compared the effects of the antibodies with a “pan-Notch inhibitor”, the gamma-secretase inhibitor (GSI). At a concentration of 5 µM, GSI inhibited ligand-dependent activation, and suppressed expression below the basal level observed in co-culture with the parental 3T3 Flp-in cells, as might be expected based on the ability of GSIs to block the activity of all Notch family members expressed on the tumor cells (Figure 4).

**Figure 5:**
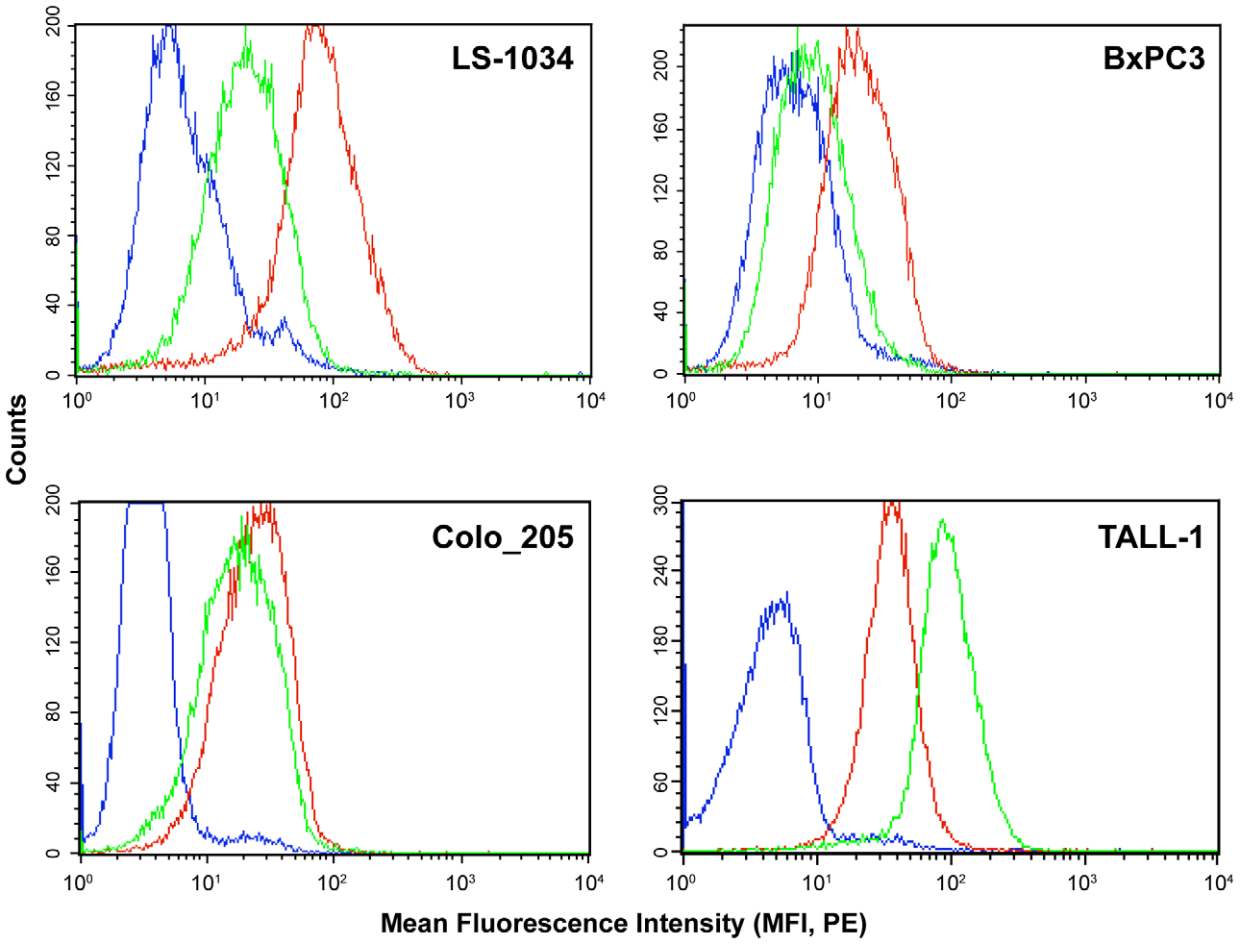
LBD and NRR antibodies bind to cancer cell lines. Notch1 surface expression in LS-1034, TALL-1, BxPC3, and Colo_205 cancer cell lines was examined by flow cytometry (FACSCalibur, BD BioSciences, San Jose, CA) after staining of cells with the LBD antibody WC613 (green line) or NRR antibody WC75 (red line), and R-PE-conjugated anti-human IgG antibody (Jackson ImmunoResearch,, Inc., West Grove, PA). An irrelevant human IgG isotype antibody (hIgG) (blue line) was used as negative control.

As described above, the potency of the antibodies in the reporter assays correlated with their affinity for Notch1, as assessed by flow cytometry conducted on cells engineered to express Notch1 stably (Table 1). To establish a correlation between phenotypic response and the binding affinity of the LBD and NRR antibodies to Notch1 expressed on the surface of cancer cells, flow cytometry was performed with LS-1034, BxPC3, Colo_205, and TALL-1 cells using saturating concentrations of the WC75 (NRR) and WC613 (LBD) antibodies. All the cell lines showed detectable levels of Notch1 on the cell surface (Figure 5) that correlated with levels of Notch1 detected in Western blots of whole cell lysates with an antibody directed against intracellular Notch1 (not shown). The relative binding affinities of NRR and LBD antibodies for Notch1 varied among cancer cell lines. With LS-1034 cells, NRR antibodies showed greater binding than LBD antibodies, while the converse was true for TALL-1 cells (Figure 5). The explanation for this cell line-dependent variation in the stoichiometry of binding of NRR and LBD antibodies is not readily apparent, and likely to be complex. It is possible, for example, that expression of various competing ligands, epitope masking by cell-type specific glycosylation, or other post-translational modifications of Notch1 [54] may differentially affect the binding of antibodies to their respective epitopes.

Notch1-dependent proliferation has been previously reported in some cancer cell lines [55]–[57]. To evaluate the anti-proliferative effect of Notch1 antibodies on a cell-line derived from a solid tumor, LS-1034 cells were grown in monolayer culture in the presence or absence of the Notch1 antibodies for up to 96 hr. Although these cell lines express wild-type Notch1 on their cell surface (Figure 5), treatment with anti-Notch1 antibodies at saturating concentrations (0.1 µM) did not affect their proliferative capacity (data not shown).

### Modulation of Ligand-Independent Notch Signaling in T-ALL Cells by NRR Antibody WC75

Leukemogenic point mutations in the NRR of Notch1 cause conformational changes that lead to ligand-independent S2 cleavage [35], suggesting that LBD antibodies should have little effect on the activation of receptors bearing such mutations. In contrast, NRR antibodies raised against wild-type receptor might be able to inhibit such mutated receptors if conformational changes induced by the mutations are not so great as to preclude antibody binding and if antibody binding prevents the adoption of conformations that are permissive for metalloprotease cleavage. To initially test this idea, Notch1 receptors bearing diverse NRR mutations were transiently expressed in U2OS cells, and antibodies were scored for their effect on activation of a Notch-dependent luciferase reporter gene. Mutations tested included six class I mutations, which destabilize the NRR; one class II mutation (from the T-ALL cell line P12-Ichikawa) consisting of a direct repeat in exon 27 of Notch1 that duplicates a 14 amino acid sequence containing the S2 cleavage site [26]; one juxtamembrane class III mutation (from the T-ALL cell line Jurkat) consisting of a direct repeat in exon 28 of Notch1 that inserts a 17 amino acid sequence [58]; and VSV, an artificial mutation that inserts 14 amino acids into the juxtamembrane region [58].

**Figure 6:**
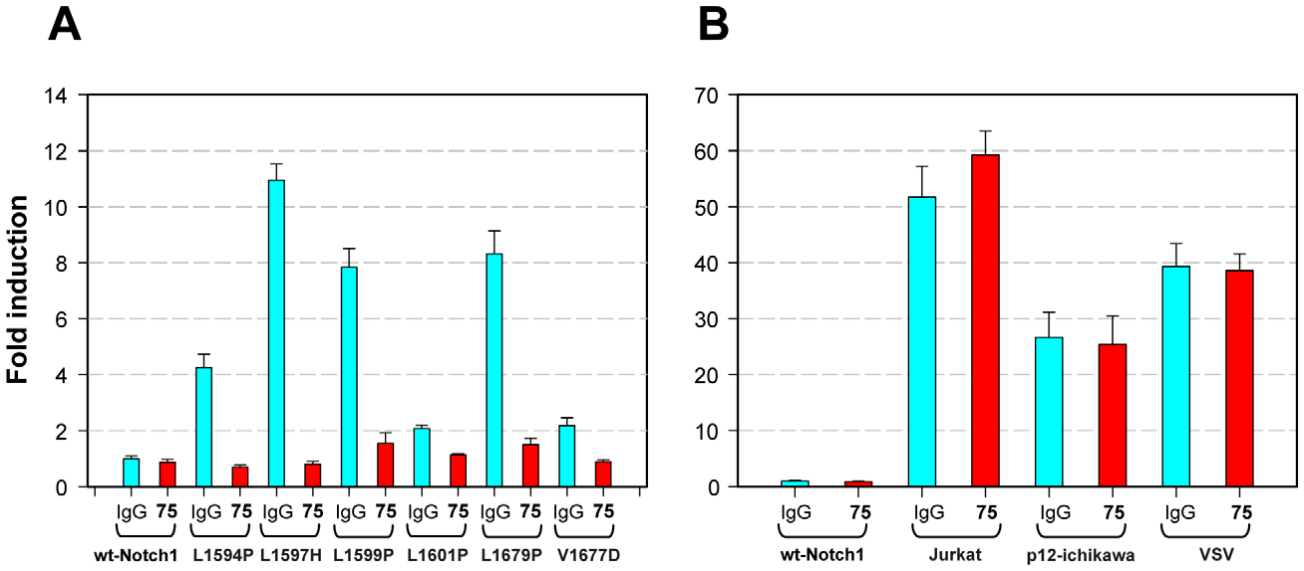
NRR antibody WC75 inhibits ligand-independent signaling by Notch1 receptors harboring T-ALL-associated mutations. T-REX-U2OS cells were transiently co-transfected with a 4xCSL-luciferase reporter construct and full-length Notch1 cDNA constructs encoding mutated receptors that exhibit ligand-independent activation of Notch signaling: (A) class I point mutations; (B) insertional mutations p12 (class II), Jurkat (class III) and VSV (juxtamembrane). Activity of luciferase after treatment with the NRR WC75 Notch1 antibody (10 µg/ml) was measured in cell lysates using the Bright-Glo assay kit (Promega). Reporter activity induced by the wt-Notch1 construct was used as baseline control. Error bars represent standard deviation.

All class I mutations tested (L1594P, L1597H, R1599P, L1601P, L1679P, V1677D) were inhibited by the NRR antibody WC75 at 10 µg/ml (Figure 6A), whereas LBD antibodies generally had little effect on these mutated forms of Notch1 (data not shown). In contrast, juxtamembrane insertional mutations (Jurkat, P12-Ichikawa, and VSV) were completely refractory to inhibition by both NRR and LBD antibodies (Figure 6B and data not shown). These data indicate that NRR antibodies are capable of recognizing and stabilizing Notch1 receptors bearing common class I mutations, and provide additional support for the idea that juxtamembranous insertional mutations activate Notch1 through a mechanism distinct from that of class I mutations [35].

**Figure 7:**
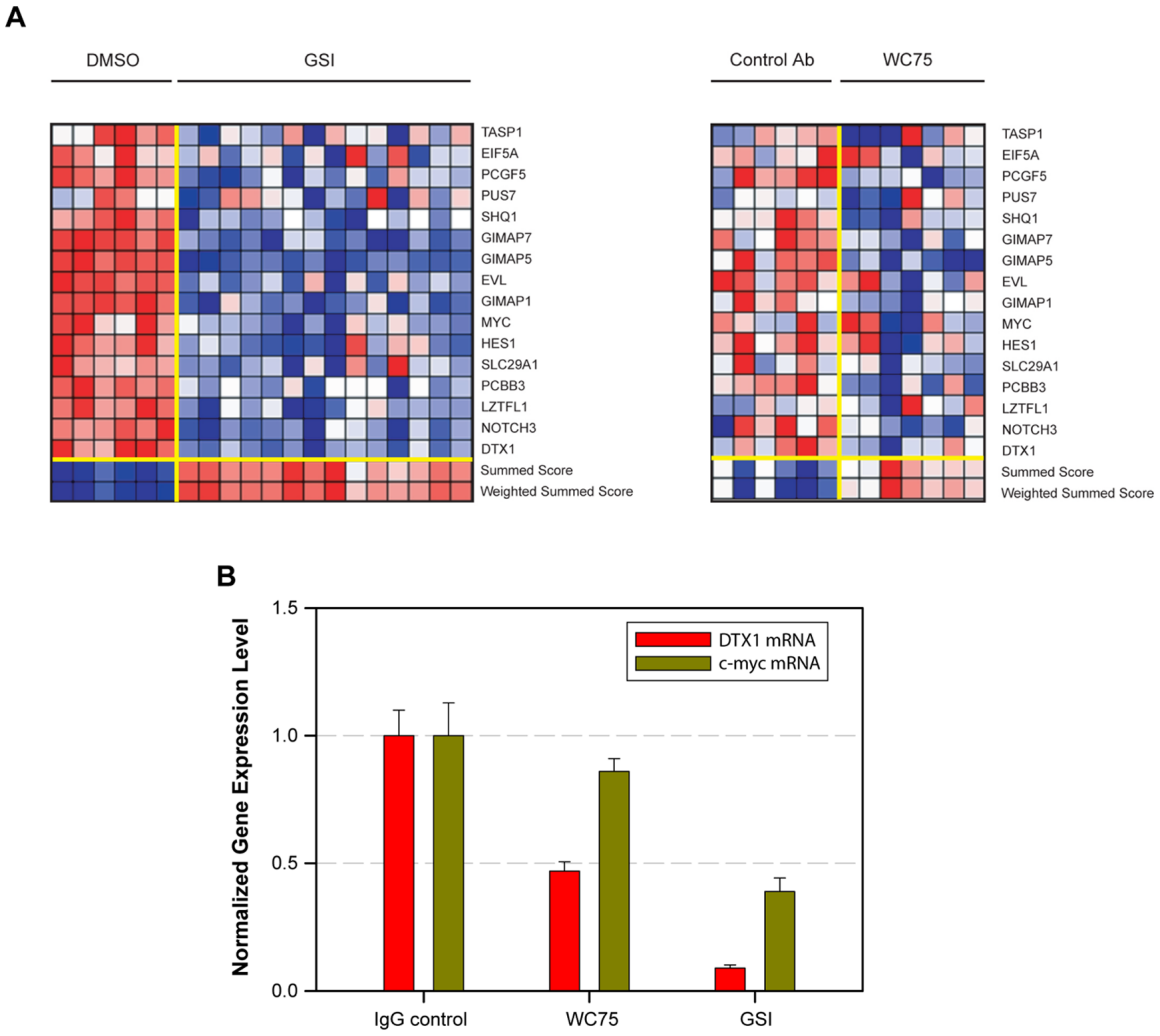
Comparison of the effects of NRR antibody WC75 or GSI on Notch1-dependent target gene expression in DND-41 cells. A) Following treatment of DND-41 cells for 72 hr with either DMSO (0.08%), GSI (compound E, 1 µM), control nonspecific human antibody (10 µg/ml), or NRR antibody WC75 (10 µg/ml), the expression levels of 20 genes that define a T-ALL-specific Notch1 signature were measured with a ligation-mediated amplification/fluorescent bead-based detection system. Each column represents an independent experimental replicate. Dark red indicates high gene expression and dark blue low gene expression. Notch marker gene expression is depicted as a ratio of the expression of the marker gene relative to the mean of four control genes. The summed score combines expression ratios by summing them with a sign determined by the expected direction of regulation as determined from the positive controls (GSI-treated). The weighted summed score metric is a variant of the summed score metric that combines expression ratios by summing them with a weight and sign determined by the signal-to-noise ratio of the positive control (GSI-treated) and negative controls (DMSO-treated). B) *DTX1* and *c-MYC* expression levels assessed by qRT-PCR following 3 days of treatment of DND-41 cells with control nonspecific human antibody (IgG, 10 µg/ml), WC75 NRR-N1 antibody (10 µg/ml), or GSI (compound E, 1 µM). Expression of each transcript was determined in triplicate, and each experiment was repeated three times.

We next asked whether the WC75 antibody could inhibit the expression of Notch1 target genes in the T-ALL cell line DND-41 which expresses Notch1 receptors bearing the compound class I mutation L1594P/D1610V. To look at the effects of WC75 on the Notch signature, we used a luminex bead-based assay that depends on ligation-mediated amplification of mRNAs captured by oligonucleotides on beads [50]. The pattern of gene expression changes induced by WC75 resembled that produced by the GSI compound E (Figure 7A), indicating that WC75 is capable of inhibiting this particular form of mutated Notch1, but the extent of inhibition by WC75 across the entire Notch1 signature was less than that produced by GSI. The relatively weak inhibitory effect of WC75 on Notch1 target gene expression was confirmed by qRT-PCR analysis of two well-characterized Notch-dependent transcripts, *DTX1* and *c-MYC* (Figure 7B).

**Figure 8:**
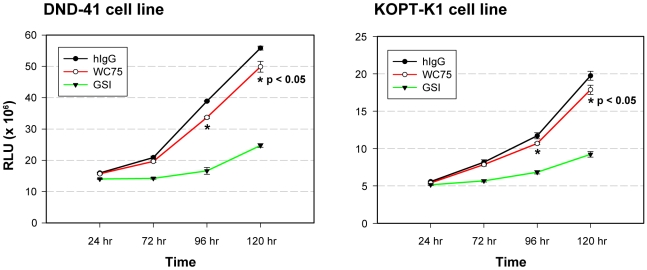
NRR WC75 Notch1 antibody proliferation of T-ALL cells. Proliferation of DND-41 (L1594P/D1610V NRR-N1 mutations) and KOPT-K1 (L1601P NRR-N1 mutation) cells (2.5×10^3^ cells/well) was assessed in a 384-well format for up to 5 days in the presence of either the WC75 antibody (10 µg/ml) or the gamma secretase inhibitor compound E (GSI, 100 nM). Growth inhibition was measured by CellTiter-Glo® (Promega).

Growth assays were also conducted to compare the effects of WC75 and GSI on T-ALL cell growth (Figure 8). WC75 reduced the growth of DND-41 cells and KOPT-K1 cells (which bear a L1601P class I NRR mutation), but to a significantly lesser degree than GSI. Isobologram studies showed that WC75 (10 µg/ml) has weakly synergistic antiproliferative effects on KOPT-K1 cells when used in combination with dexamethasone (combination index = 0.45, not shown), whereas GSI produced stronger synergistic effects (combination index = 0.1, not shown). Taken together, these studies show that in T-ALL cells, signals generated by Notch1 receptors bearing class I NRR mutations are not inhibited as effectively by NRR antibodies as they are by GSI.

## Discussion

Notch pathway inhibitors represent an opportunity for targeted treatment of several different human cancers. Tumors for which inhibition of Notch signaling may be particularly desirable include breast cancer, where high levels of Notch1 signaling are associated with poorer prognosis [28], [31], and T-ALL, in which activating mutations in Notch1 are found frequently and where treatment with Notch pathway inhibitors, such as GSIs, arrests growth [34], [40], [41].

In the studies reported here, we characterize the *in vitro* activity of Notch1 monoclonal antibodies derived from cell-based and solid-phase screening of a phage display library. Antibodies could be grouped into two mechanistically distinct classes, ligand-competitive antibodies targeting the EGF repeat 1–13 region and allosteric, NRR-binding antibodies. Antibodies in both groups have potencies in the nanomolar to picomolar range and are highly specific for Notch1. The antibodies recognize endogenous receptors on tumor cell lines, inhibit the expression of Notch target genes in some tumor cell lines, and block Notch-dependent transcription in transfected cells. Ligand-competitive antibodies bind to the EGF-repeat 1–13 region (LBD) of the receptor and show a strong dependence on the particular ligand-expressing cell line used for co-culture. The variation in antagonist potency as a function of activating ligand might arise for a number of different reasons. Possibilities include not only differences in the intrinsic affinity of Notch1 for various ligands, but also variation in ligand expression level, differential modulation of ligand affinity by glycosyltransferase modification of Notch1, variable ligand-mediated cis-inhibition in Notch-expressing cells, etc. Additional mechanistic studies will be required to evaluate the potential therapeutic use of these ligand-competitive antibodies. For instance, DLL4-dependent events, such as tumor neoangiogenesis [33], may be relatively insensitive to the LBD antibodies reported here. On the other hand, cancers in which over-expression of Jag1, Jag2, and DLL1 are associated with poor survival, such as prostate [60] and breast [61]–[63] carcinomas, CNS tumors [57], and multiple myeloma [62], may be tractable targets.

The second group of inhibitory antibodies binds to the NRR, the activation switch of the receptor located ∼1000 residues C-terminal to the ligand-binding EGF repeats. The mechanism of inhibition of NRR antibodies with respect to ligands appears to be allosteric, with little dependence on the type of ligand used for transactivation. However, the NRR antibodies were incapable of completely inhibiting ligand-dependent Notch1 activation; whether this stems from masking of the binding epitope in a subset of receptors, residual intrinsic responsiveness of antibody-bound receptors, or some other mechanism remains to be determined. Of note, binding of the WC75 and WC629 NRR antibodies is abrogated by EDTA (Supplemental Figure S1), which relaxes the structure of the NRR [3], [59]. Together these data indicate that NRR antibodies bind to a conformational epitope on the auto-inhibited conformation of the NRR and prevent adoption of the open, protease-accessible conformation upon ligand interaction.

Cell culture studies with human solid tumor cell lines, including LS-1034, showed that the LBD and NRR antibodies have no significant anti-proliferative effect. The lack of anti-proliferative activity in monolayer culture is not unexpected, as even GSIs lack activity against many solid tumor cells in culture, despite their activity in *in vivo* models (unpublished data). Growth inhibition and apoptosis have been reported following siRNA mediated knock-down of Notch1 [55], [57], [65]. It is possible that down-regulation of Notch protein levels may have a greater impact than inhibition of ICN1 production on cross-talk with other pathways that drive cancer growth [66], [67], as well as the expression of key factors involved in cell cycle progression [68]. Cell culture models of physiologically relevant Notch-ligand interactions have been reported [55], [57]; however, *in vivo* models will be required to conclusively evaluate the therapeutic potential of Notch1 antibodies.

Of interest, the NRR antibodies bind and inhibit ligand-independent activation of Notch1 receptors harboring T-ALL associated mutations, while LBD antibodies generally do not. Nevertheless, it appears that in contrast to GSIs, the ability of NRR antibodies to inhibit growth is likely to be limited to T-ALL lines bearing class I Notch1 mutations, as receptors harboring unusual juxtamembrane insertional mutations [35], [58] were completely resistant to the inhibition by NRR antibodies. In addition, even Notch1 receptors harboring class I mutations appear to be partially resistant to inhibition, particularly in T-ALL cells. In part, this may be due to the allosteric mechanism of inhibition by NRR antibodies which, as shown for wild-type Notch1 signaling, results in incomplete inhibition. An additional possibility is that aberrant trafficking of such receptors in T-ALL results in intracellular proteolysis and activation in vesicular compartments that are not accessible to antibody, but can be reached by membrane-permeable GSIs.

Similar to the results with human solid tumor cell lines expressing wild-type Notch1, the proliferation of T-ALL cell lines was minimally affected by NRR antibodies. However, T-ALL cell lines are significantly more sensitive to GSI-mediated inhibition of Notch1 activation. Together these data suggest that the therapeutic potential of NRR antibodies is higher in tumors that have intact extracellular Notch1 and depend on ligand for Notch1 activation; breast cancer is one such tumor. It is also possible that such anti-Notch antibodies may have value as inhibitors of stromal activities that support tumor cell growth, such as angiogenesis, which depends on a DLL4-Notch1 signaling axis [32], [69]. In addition to their therapeutic potential, these antibodies may find utility as biomarker tools, for detection of Notch1 on the surface of tumor cells, and as probes of Notch1 function and signaling mechanisms.

## Supporting Information

Figure S1 Calcium-dependence of epitope binding by anti-NRR antibodies. Biotinylated Notch1 NRR was captured onto neutravidin-coated 96-well plates. Binding of the NRR antibodies was allowed to proceed for one hour in Tris buffer (25 mM, pH 7.4), containing NaCl (150 mM), CaCl2 (5 mM), 0.05% Tween, and 0.5% BSA. The (−) column for each condition indicates the absence of EDTA, and the (+) column indicates the presence of EDTA (10 mM). Antibody binding was detected with a goat anti-human antibody conjugated to horseradish peroxidase using the fluorogenic substrate quantaBlu (Pierce-Thermo). The three control experiments were performed by omitting the Notch1 NRR antigen (no Notch1 NRR), the anti-Notch1 NRR (no 1-Ab), or the secondary anti-human antibody (no 2-Ab).
(2.04 MB TIF)

Figure S2 Antibodies WC75 (A) and WC613 (B) immunoprecipitate Notch1 but not Notch4. 293T cells were transfected with plasmids expressing the complete ectodomains of Notch1 or Notch4 containing His6-tags at their C-terminal ends. Immunoprecipitation was performed after lysis of the transfected cells. WCE: whole cell extracts; FT: supernatant remaining after immunoprecipitation; IP: WC75 (A) or WC613 (B) immunoprecipitate. Detection was performed with an anti-His6 antibody. His6-tagged molecular weight markers are loaded in the leftmost lane.
(0.44 MB TIF)
