# Supplementary figures and images for: Characterization of Notch1 Antibodies That Inhibit Signaling of Both Normal and Mutated Notch1 Receptors
Source: PLoS One. 2010 Feb 8;5(2):e9094. doi: 10.1371/journal.pone.0009094 (PMC2817004; doi:10.1371/journal.pone.0009094)

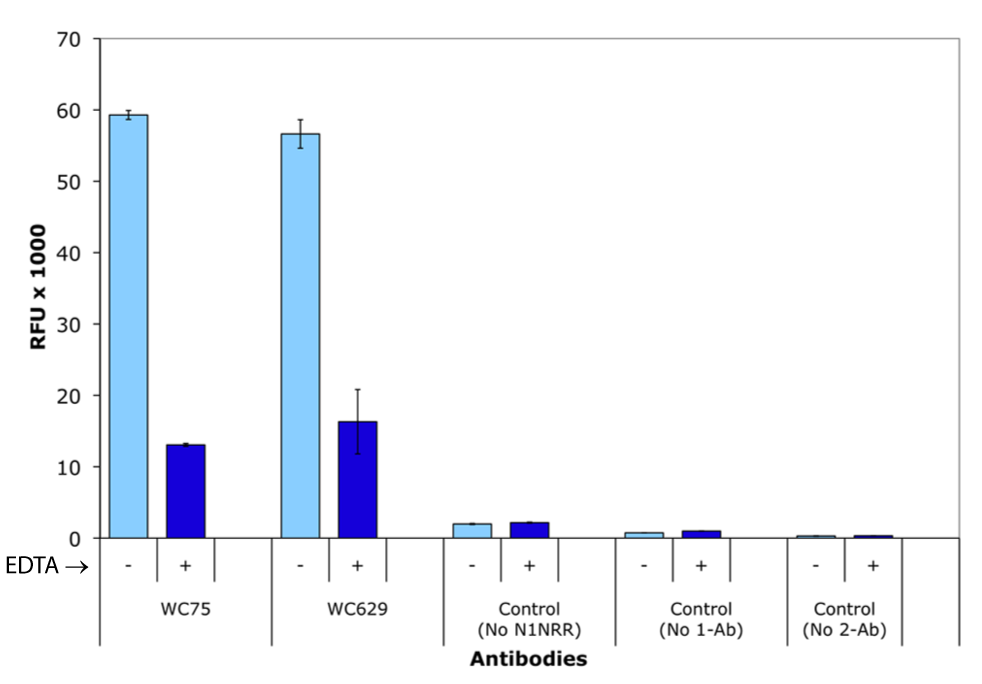

Supplement: Figure S1 — Calcium-dependence of epitope binding by anti-NRR antibodies. Biotinylated Notch1 NRR was captured onto neutravidin-coated 96-well plates. Binding of the NRR antibodies was allowed to proceed for one hour in Tris buffer (25 mM, pH 7.4), containing NaCl (150 mM), CaCl2 (5 mM), 0.05% Tween, and 0.5% BSA. The (−) column for each condition indicates the absence of EDTA, and the (+) column indicates the presence of EDTA (10 mM). Antibody binding was detected with a goat anti-human antibody conjugated to horseradish peroxidase using the fluorogenic substrate quantaBlu (Pierce-Thermo). The three control experiments were performed by omitting the Notch1 NRR antigen (no Notch1 NRR), the anti-Notch1 NRR (no 1-Ab), or the secondary anti-human antibody (no 2-Ab). (2.04 MB TIF) [file pone.0009094.s001.tif]

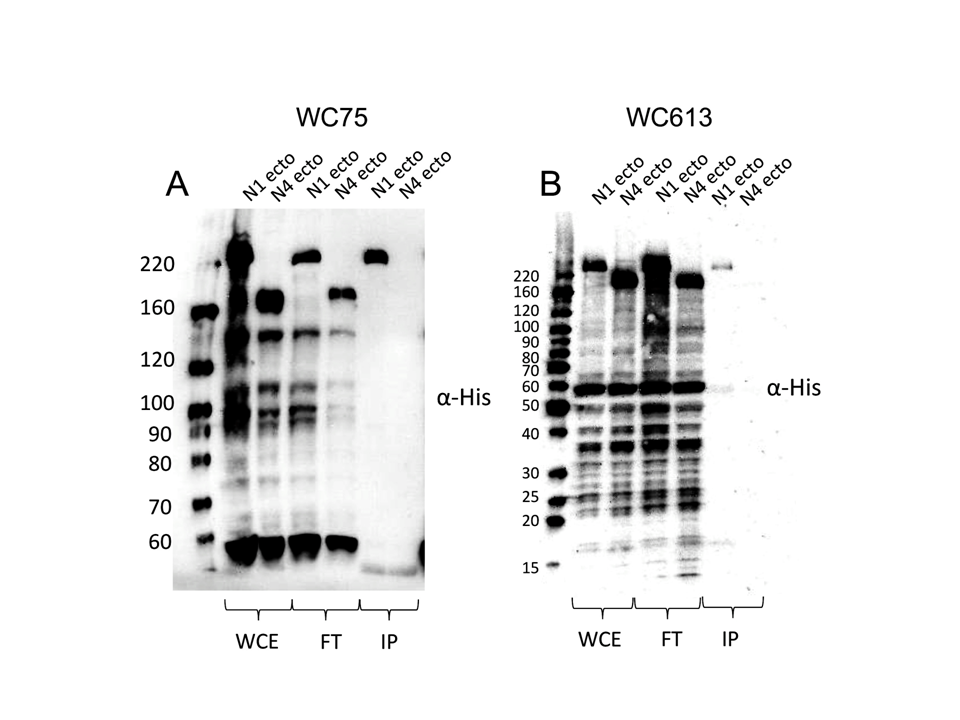

Supplement: Figure S2 — Antibodies WC75 (A) and WC613 (B) immunoprecipitate Notch1 but not Notch4. 293T cells were transfected with plasmids expressing the complete ectodomains of Notch1 or Notch4 containing His6-tags at their C-terminal ends. Immunoprecipitation was performed after lysis of the transfected cells. WCE: whole cell extracts; FT: supernatant remaining after immunoprecipitation; IP: WC75 (A) or WC613 (B) immunoprecipitate. Detection was performed with an anti-His6 antibody. His6-tagged molecular weight markers are loaded in the leftmost lane. (0.44 MB TIF) [file pone.0009094.s002.tif]
